# Cardiac, Hepatic and Renal Dysfunction and *IL-18* Polymorphism in Breast, Colorectal, and Prostate Cancer Patients

**DOI:** 10.31557/APJCP.2021.22.1.131

**Published:** 2021-01

**Authors:** Govand Qader, Mukhlis Aali, Shukur W Smail, Kazhan Mahmood, Bestoon Hasan, Karwan M-Amen, Dlzar Bayz Rahman, Fikry A Qadir, Dara K Mohammad, Hastyar H Najmuldeen, Fryad Majeed Rahman, Seepal Ibrahim Ahmad, Nergz S Salih, Zainab M Khdhr, Bushra A Mohammed, Asuda M Majeed, Xanda M Hasan, Bushra H Khidhir, Eman S Muhammad, Bahar A Muhamadsalih, Simav K Hasan, Aram J Hamad, Zahra K Esmail, Chra M Ismael, Shan M Husaen, Chiavan A Abdulla, Bashdar M Hussen, Zjwan Housein, Mudhir Shekha, Abbas Salihi

**Affiliations:** 1 *Department of Biology, College of Science, Salahaddin University-Erbil, Kurdistan Region, Iraq. *; 2 *Department of Medical Analysis, Faculty of Science, Tishk International University, Erbil, Iraq. *; 3 *Department of Midwifery, College of Nursing, Hawler Medical University, Erbil, Kurdistan Region, Iraq. *; 4 *Department of Cancer Registry, Cancer Control Unit, Erbil Directorate of Health, Erbil, Iraq. *; 5 *Department of Nursing, College of Nursing, Hawler Medical University, Erbil, Kurdistan Region, Iraq. *; 6 *Internal Laboratory, Hawler Teaching Hospital, Erbil Directorate of Health, Erbil, Iraq. *; 7 *College of Agricultural Engineering Sciences, Salahaddin University-Erbil, Erbil, Kurdistan Region, Iraq. *; 8 *Center for Hematology and Regenerative Medicine (HERM), Department of Medicine Huddinge, Karolinska Institutet, 141 83 Stockholm, Sweden. *; 9 *Department of Biology, College of Science, University of Sulaimani, Kurdistan Region, Iraq. *; 10 *Medical Laboratory Analysis, Cihan University-Sulaimaniya, Slemani, Iraq. *; 11 *Emergency Hospital, Duhok General Health Directorate, Duhok, Kurdistan Region, Iraq. *; 12 *Department of Pathological Analysis, Faculty of Science, University of Knowledge, Erbil, Kurdistan Region, Iraq. *; 13 *College of Pharmacy, Hawler Medical University, Kurdistan Region, Iraq. *; 14 *Department of Medical Laboratory Technology, Health Technical College, Erbil Polytechnic University, Erbil, Iraq. *

**Keywords:** Breast cancer, colorectal cancer, prostate cancer, tumor markers- interleukin-18 polymorphism

## Abstract

**Introduction:**

The present study aimed to determine the alterations in the serum levels of tumor markers used to evaluate cardiac, renal and liver function, and detect the *interleukin (IL)-18 rs1946518* polymorphism in breast (BC), colorectal (CRC) and prostate cancer (PCa) patients.

**Methods:**

Blood samples were collected from 65 female BC, 116 CRC, 79 PCa and 88 myocardial infarction (MI) patients, and 110 healthy individuals to determine the concentration of tumor and cardiac markers. Furthermore, the *IL-18 rs1946518* polymorphism was assessed using amplification refractory mutation system (ARMS)-PCR.

**Results:**

The serum levels of the tumor markers cancer antigen 15-3 (CA 15-3), carbohydrate antigen 19-9 (CA 19-9), carcinoembryonic antigen (CEA) and total prostate-specific antigen (TPSA) were significantly increased in cancer patients compared with healthy controls. Furthermore, the activity of high-sensitivity cardiac troponin T (hs-cTnT) and creatine kinasemyocardial band (CK-MB) was enhanced in MI patients, however, their activity was unchanged in cancer patients. The activity of alkaline phosphatase (ALP), and the serum concentration of aspartate aminotransferase (AST), alanine aminotransferase (ALT) and urea were markedly elevated in CRC and PCa patients, respectively, compared with the control group. Although, no significant differences were observed in the -607 C/A polymorphism and allele frequency of IL-18 among BC, CRC patients and healthy individuals, the odds ratio (OR) was 1.75 for both C and A allele in BC patients. Therefore, the -607 C/A polymorphism could be considered as a risk factor for BC.

**Conclusion:**

The aforementioned results suggested that tumor markers could be considered as excellent biomarkers for the early detection of BC, CRC and PCa, whereas the concentration of liver enzymes could serve as an alternative indicator for the diagnosis of CRC and PCa. Additionally, the rs1946518 polymorphism in the *IL-18* gene could be considered as a risk factor for the occurrence of BC, CRC and PCa.

## Introduction

Cancer is the leading cause of death in high-income countries and the second leading cause in low- and middle-income countries. It has been estimated that approximately 1 to 4 deaths in the United States are attributed to cancer (Brown et al., 2012). Breast cancer (BC) is the most common malignant tumor and foremost cause of death in women globally (Youlden et al., 2014). Likewise, prostate cancer (PCa) is one of the most common tumors in men, while colorectal cancer (CRC) is considered the third most commonly diagnosed type of cancer worldwide, with approximately 1.23 million new cases each year (Ning et al., 2018)).

Nowadays, several biomarkers are available for the early detection and progressive stages of different types of cancer using blood samples, including total the prostate-specific antigen (TPSA) for the diagnosis of PCa, the carcinoembryonic antigen (CEA) for gastrointestinal, breast and lung cancer, the cancer antigen 125 (CA 125) for ovarian cancer, the carbohydrate antigen 19-9 (CA 19-9) for CRC and pancreatic cancer, and the CA 15-3 for BC (Diamandis, 2014). Since no cure is available for many types of cancer, patients eventually progressing to metastatic cancer, which affects other organs such as the liver, kidney, heart and lungs, with fatal consequences for the patients (Arends et al., 2017). Such organ failure may cause more deaths than cancer itself (Frères et al., 2018). Therefore, biomarkers are widely used to detect cancer at an early stage and have been extensively investigated in several studies (Cao and Wang, 2012). Furthermore, the risk for cardiac diseases has been regarded as an important barrier for cancer therapies, as their mortality rate is higher compared with that of cancer (Frères et al., 2018). For the majority of tumors, the cancer cell metastasis from the primary tumor to surrounding tissues and to distant vital organs is responsible for the majority of cancer-related deaths, accounting for about 90% of cancer cases (Akinyemiju et al., 2018). Therefore, the early detection of circulating biomarkers could be considered as a highly valuable tool for cancer treatment (Marcuello et al., 2019).

Interleukin (IL)-18, a member of the IL-1 family, activates the immune cells involved in both innate and adaptive immune responses, thus increasing the immune defense against tumor cells. It has been reported that IL-18 exerts a significant role in tumor progression in several types of cancer. The *IL-18* gene expression and serum levels have been shown to be elevated in blood of collected from metastatic patients compared with patients without metastasis and healthy individuals (Park et al., 2007). Additionally, it has been suggested that IL-18 modulates the immune system for attacking cancer cells via inhibiting tumor growth and angiogenesis in ovarian cancer, attenuating cell proliferation and invasion ability in BC, and increasing the cytotoxic effects of the chemotherapeutic drugs on colon malignant cells (Jia et al., 2016). Several studies have demonstrated the association between various types of cancer and polymorphisms on the *IL-18 *gene, despite the chronic inflammatory status of cancer patients (Hosseini-Baraftabi et al., 2019; Li et al., 2019).

A previous study from our lab, has revealed that the number of patients with cancer is increasing in the Kurdistan region of Iraq (Qader et al., 2020), however, gaps still exists between Kurdistan and the rest of the world in terms of number and quality of cancer-related publications. Therefore, the present study aimed to identify and determine the changes in the levels of the most effective and appropriate biomarkers used for the early diagnosis of different types of cancer and cardiac diseases, and to further investigate the possible association between a polymorphism at position -607 C/A (rs1946518) of the *IL-18* gene and BC, CRC and PCa Kurdish patients.

## Materials and Methods

*Patients*

 A total of 458 individuals were enrolled in the current study. Among them, 65 were diagnosed with BC, 116 with CRC, 79 with PCa and 88 with myocardial infarction (MI). Concurrently, 110 healthy volunteers were also included in the study. Participants were recruited from Erbil city hospitals. The present study was authorized and approved by the Human Ethics Committee of the College of Science, Salahaddin University, Erbil. All patients and healthy volunteers provided written informed consent for the publication of their data in the current study.

*Blood collection*

Blood samples were obtained by phlebotomy under aseptic conditions. Venous blood was aspirated with a 5 ml syringe, collected in both plain and anticoagulant tubes, and maintained at room temperature. Subsequently, serum was separated from whole blood, placed in microcentrifuge tubes, and stored in a freezer at -60°C until used. Blood in the anticoagulant tubes was used for subsequent molecular diagnostic analyses based on PCR.

*Estimation of tumor and cardiac markers*

 The concentration of CA 15-3, CEA, CA 19-9, TPSA, hs-cTnT, and CK-MB were determined using the Cobas e411 analyzer (Roche Diagnostics GmbH) and a ready-to-use reagent kit in individual cassettes, according to the manufacturer’s instructions. This detection method relies on the measurement of immunoreactivity utilizing electrochemiluminescence.

*Renal and liver function tests*

The serum concentration of creatinine and urea, as well as the activity of aspartate transaminase (AST), alanine aminotransferase (ALT), and alkaline phosphatase (ALP) were measured using the Bt35i and BioLIS 50i automated clinical analyzers (Tokyo Boeki Medisys Inc.). This detection method relies on the use of a spectrophotometer.

*Amplification refractory mutation system (ARMS)-PCR*

Genomic DNA was extracted from whole blood, as previously described (Hashemi et al., 2017). The following primer sequences were used for amplifying the IL-18 polymorphism: forward outer primer, 5’-CCTACAATGTTACAACACTTAAAAT-3’ 

and reverse outer primer,

5’-ATAAGCCCTAAATATATGTATCCTTA-3’;

and forward inner primer, 

5’- GATACCATCATTAGAATTTTGTG-3’ and reverse inner primer, 5’- GCAGAAAGTGTAAAAATTATCAA-3’. The genotyping of the IL-18 -607 C/A polymorphism was assessed using the tetra-primer ARMS (T-ARMS) method, as previously described (Taheri et al., 2012). Furthermore, PCR was performed using a commercially available PCR premix (Genet Bio), according to the manufacturer’s recommendations. The PCR reaction was performed in a total volume of 20 µl containing 10 µl premix solution, 3 µl DNA template (concentration, ~100 ng/µl), 1 µl from each primer (10 µM) and 3 µl nuclease-free water. The PCR conditions were as follows: initial denaturation at 95°C for 5 min followed by 30 cycles, each consisting of 30 sec at 95°C (denaturation), 20 sec at 54°C (annealing), and 30 sec at 72°C (extension), with a final extension at 72°C for 10 min. The PCR products were analyzed by electrophoresis on a 2% agarose gel supplemented with 0.5 µg/ml ethidium bromide and were then visualized under ultraviolet light. Product sizes were 208 bp for the C allele, 278 bp for the A allele, and 440 bp for the control band.

*Statistical analysis*

The statistical analysis for the serological data and the comparison between the tumor and control groups were performed using the Mann-Whitney U test (P<0.05). Cardiac function data were compared with those of the control and MI groups using the Kruskal-Wallis test. Normality tests, namely D’Agostino and Pearson omnibus, Shapiro-Wilk and KS normality tests, were carried out for all data. The area under the curves (AUC) was calculated using the receiver operating characteristic (ROC) curve. All values were expressed as median and range, and P<0.05 was considered to indicate a statistically significant difference. All statistical analyses, calculations and graphs were performed using GraphPad Prism 6.0 software (GraphPad Software, Inc.).

## Results

*Tumor markers between cancer patients and healthy controls*

The serum levels of CA15-3, CA19-9 and CEA in BC and CRC, and TPSA in PCa patients (P<0.001) were significantly increased compared with those noted to healthy individuals (Table 1).

*Levels of cardiac markers between healthy controls, and cancer and MI patients*

The activity of hs-cTnT (ng/ml) and CK-MB was notably elevated in MI patients compared with healthy controls, and BC, CRC and PCa patients (P<0.001). However, there was no statistically significant difference in hs-cTnT and CK-MB levels between BC, CRC and PCa patients, and healthy volunteers (Table 2).

*Liver and renal function tests between healthy controls and cancer patients*

The activity of AST, ALT and ALP, and the serum concentration of urea and creatinine were unchanged in BC patients, whereas ALP was significantly increased in CRC patients compared with healthy individuals (P<0.001). Furthermore, the activity of AST and ALT, and urea serum levels were significantly increased in PCa patients compared with the control group (Table 3).

*Selection of the biomarkers*

**Figure 1:**
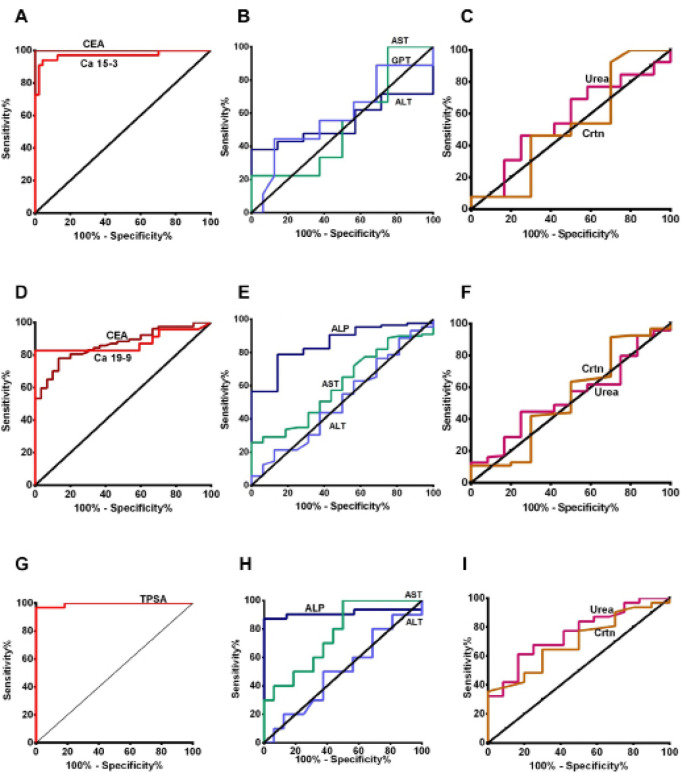
ROC Curve for the Level of Serum Tumour Markers, Liver and Renal Function Tests in BC, CRC and PCa Patients. A, B, C represent Ca15-3, CEA, liver and renal function tests in BC patients, respectively. D, E, F represent Ca19-9, CEA, liver and renal function tests in CRC patients, respectively. G, H, I represent TPSA, liver and renal function tests in PCa patients, respectively

 As shown in Figure 1, CEA and CA15-3 were the most efficient biomarkers for the early detection of BC, with AUC values of 1 and 0.97, respectively. In addition, CA 19-9, CEA and ALP were the most efficient biomarkers for CRC (AUC, 0.87, 0.86 and 0.85, respectively), and TSPA and ALP for PCa (AUC, 0.99 and 0.91, respectively). Furthermore, urea, creatinine and AST were considered as good indicators for detecting PCa, with AUC values of 0.75, 0.7 and 0.76, respectively.

*Genotypes and allele distribution of the IL-18 polymorphism*

*The distributions *of the genotype and allele frequencies of the IL18-607 C/A polymorphism in patients and healthy groups are shown in Table 4. The results showed that 38.88% of BC patients exhibited the homozygous CC genotype, 50% the heterozygous CA genotype and 11.22% the homozygous AA genotype. In addition, 18 and 82% of CRC patients presented the homozygous CC and heterozygous CA genotype, respectively. The genotype rates of the -607 C/A polymorphism in healthy individuals were 46, 54 and 0.0% for CC, CA and AA genotype, respectively. However, no significant differences were observed in the distribution of the -607 C/A polymorphism of the *IL-18* gene among BC and CRC patients, and healthy individuals (P>0.05). As shown in Table 4, the C allele frequency was 61% in BC and 59% in CRC patients, while the A allele frequency was 39 and 41%, respectively. In healthy individuals, the C and A allele frequencies were 73 and 27%, respectively. 

There were no significant differences in the allele frequency between patients and healthy controls (P>0.05). Additionally, the odds ratio (OR) value was 1.75 for both C and A allele in BC patients. Therefore, the -607 C/A polymorphism could be considered as a risk factor for BC. Furthermore, the frequency rates of homozygous CC, heterozygous CA and homozygous AA genotypes in BC and PCa patients were 24.1, 61.1 and 14.8%, respectively. In addition, the. CC and CA genotype frequency rates were 46 and 54% in healthy subjects, respectively, whereas the AA genotype was not detected. Furthermore, the IL18 -607 C/A AA genotype was associated with a significantly increased risk for PCa compared with the IL18 -607 C/A CC genotype [P=0.001, OR=0.033, 95% confidence interval (CI)=0.002-0.633)]. Additionally, a significant association was identified for the recessive model (CC vs. AA/CA: OR=0.372, 95% CI=0.161-0.858, P=0.024). However, no significant association was observed in the heterozygote model (CA vs CC: OR=0.462, 95% CI=0.197-1.081, P=0.092). Finally, the frequency rate of the carrier for the mutant A allele was significantly higher in PCa patients compared with healthy volunteers, and a statistically significant difference was observed in the frequency rates between the two IL-18 alleles (C vs A: OR=0.445, 95% CI=0.248-0.796, P=0.006).

**Table 1:** Comparison between Serum Levels of Ca15-3 (U/ml) and CEA (ng/mL), Ca19-9 (U/ml) and CEA (ng/ml) and TSPA (U/ml) in Control, Breast, Colorectal and Prostate Cancer Patients

	Control	BC	CRC	PCa
Ca15-3	19.47 (13.38-24.07)	52.63 (35.28,130.8)***		
CEA	1.57 (0.97-2.13)	15.6 (6.74-52.75)***		
Ca19-9	5.77 (1.79-6.41)		19.37(9.18-46.16)***	
CEA	1.105 (0.57-1.59)		3.1 (1.88-22)***	
TPSA	1.26 (0.62-2.98)			64.01 (18.93-165.7)***

**, represents P value 0.01; ***, represents P value 0.001

**Table 2:** Comparison between Serum Levels of hs-TnT (ng/ml) and CK-MB (IU) in Control, MI, Breast, Colorectal and Prostate Cancer Patients

	Control	MI	BC	CRC	PCa
hs-TnT	0.005 (0.004-0.008)***	0.034 (0.02-0.086)	0.003 (0.003-0.003)***	0.003 (0.003,0.0055) ***	0.003 (0.003-0.0055)***
CK-MB	16.3 (14, 18.83)**	38.4 (28.7, 80.35)	22.65 (14.73, 26.38)**	15.14 (7.21-43.91)***	6.82 (5.86, 12.3)***

**, represents P value 0.01; ***, represents P value 0.001

**Table 3:** Comparison between Renal and Liver Function Tests in Control, Breast, Colorectal, and Prostate Cancer Patients

	Control	BC	CRC	PCa
AST	21.55 (15.8, 28.83)	21 (17,29. 5)	24 (19, 35)	27.9 (22.53,39.25)*
ALT	17.1 (11.33, 31.75)	15 (9.5, 26)	18 (12, 29.3)	16.25 (11.38, 29.05)
ALP	89.9 (65.4, 112)	84 (35.44, 202.2)	198 (118.4, 269)***	291 (224,733.4)***
Urea	34.15 (28.8, 39.6)	36.46 (32.5, 41)	34 (26.8, 39)	44 (37, 61)**
Crtn	0.85 (0.6, 1.23)	0.89 (0.76, 1.11)	0.87 (0.75, 1.05)	1.08 (0.81, 1.56)

*, represents P value 0.05; **, represents P value 0.01; ***, represents P value 0.001

**Table 4:** The Genotypes and Allele Distribution of IL-18 Polymorphism in Control, Breast, Colorectal, and Prostate Cancer Patients

Polymorphism	Control	BC				CRC				PCa			
		%	OR	95% Cl	P value	%	OR	95% Cl	P value	%	OR	95% Cl	P value
CC	46	38.88	1	-	-	18	1	-	-	24.1	1	-	
CA	54	50	1.13	0.27-4.64	0.87	82	3.98	0.63-24.74	0.131	61.1	0.46	0.2 to 1.08	0.092
AA	0	11.22	5	0.2-122.8	0.18	-	-	-	-	14.8	0.03	0.002 to 0.63	0.001
AA+CA	54	61.22	1.38	0.34-5.51	0.65	-	-	-	-	75.9	0.37	0.16 to 0.86	0.024
C	73	61	1.75	0.61-5.01	0.29	59	1.9	0.59-6.16	0.28	54.6	0.45	0.248 to 0.8	0.006
A	27	39				41				45.4			

## Discussion

The results of the present study indicated that the tumor markers CA 15-3, CA 19-9 and TPSA were the best biomarkers for detecting BC, CRC and PCa, respectively. In addition, the results revealed that CEA could serve as an excellent general tumor biomarker, and especially for CRC. Consistent with our findings, a meta-analysis from China confirmed the association between the CA 15-3 serum levels with BC susceptibility (Fu and Li, 2016). On the other hand, it has been reported that the serum levels of the CA15-3 and CEA biomarkers may be used for acquiring sufficient information about the disease and its progression status, when tissue specimens are not available (Li et al., 2019). Furthermore, a previous study has suggested that the increased preoperative serum CA 19-9 levels are considered as a prognostic factor for CRC (Shin et al., 2019). Additionally, CEA is positively correlated with CRC stage and patient’s age, while its levels are significantly increased in male patients (Ng et al., 2017). A recent study has suggested that initial patient evaluation and treatment decisions are based on a risk stratification scheme that incorporates the three most important prognostic biomarkers at diagnosis: the clinical stage, biopsy Gleason grade/score, and serum PSA (Gaudreau et al., 2016). 

PSA is the most widely used biomarker for the early detection of PCa. Other researchers have suggested that CA15-3 alone is not sufficient for the early detection of BC and its nodal status and grade, however, they accept that it may be considered as a pre-diagnostic marker for more aggressive phenotypes (Kazarian et al., 2017). Morita et al demonstrated that there was no difference in the diagnostic value of CA 19-9 and CEA regarding the early detection of CRC. In addition, they hypothesized that CA 19-9 could not be used to predict the prognosis or relapse of CRC. Correspondingly, the authors showed that the sensitivity of serum CA19-9 was low, therefore, they did not recommend the addition of serum CA19-9 biomarker to the current standard surveillance strategies (Okamura et al., 2017). PSA has several limitations as a biomarker, as it is not able to well distinguish between prostate cancer and benign prostatic hyperplasia or between indolent and aggressive prostate cancers (Romero Otero et al., 2014).

Furthermore, the results of the present study demonstrated that the hs-cTnT and CK-MB biomarkers were significantly elevated in MI individuals, but not in BC, CRC and PCa patients compared with healthy volunteers. Therefore, the high specificity and selectivity of methods that are based on the detection of troponins led to the inclusion of troponin levels into the criteria for the diagnosis of MI (Adamcova et al., 2016). In addition, it has been suggested that there is no significant risk of cardiac failure in BC patients nor healthy individuals, when the levels of CK-MB are reduced (Blaes et al., 2015). However, no association has been documented between the survival rate and elevated hs-cTnT levels in BC and PCa patients (Florido et al., 2019).

Further studies on liver biomarkers have revealed that ALP may be considered as a valuable and excellent hepatic biomarker for the early detection of BC, CRC and PCa. Liver injury and the resulting biochemical abnormalities are very common in cancer patients, may be due to metastasis, concomitant drugs, or previous liver damage, such as cirrhosis (Field et al., 2008). ALP has been found to be a valuable tumor marker with high specificity in detecting CRC (Saif et al., 2005), PCa (Rao et al., 2017) and metastatic and different stages of BC (Singh et al., 2013). However, a study demonstrated that CA15-3 was a better indicator for predicting BC relapse than ALP, while the combination of both biomarkers exhibited the best prediction results on BC relapse (Keshaviah et al., 2007). Furthermore, ALP velocity appears to be a strong independent predictor of bone metastases and overall survival in men with increased PSA levels (Metwalli et al., 2014).

The expression of *IL-18* has been previously investigated in several types of cancer, including gastric and colon carcinomas. In colon adenocarcinomas, reduced or abolished IL-18 synthesis levels have been detected, thus suggesting that IL-18 may play a tumor-suppressive role (Cao et al., 1999). Only one previous study has investigated the association between the IL-18 -607 A/C polymorphism and malignancy. In this study, no association between this specific polymorphism and oral cancer was detected (Jia et al., 2016). Genetic alterations such as single nucleotide polymorphisms are considered as major risk factors for BC (Zhao et al., 2012). 

The present study aimed to investigate the association between an IL-18 polymorphism and BC, CRC and PCa. It has been reported that the -607 A/C polymorphism affects the transcription of the *IL-18* gene in patients with CRC and healthy controls. The results of the current study revealed that A/C heterozygotes exhibited an increased risk for CRC, while homozygotes for the highly presented C allele were protected against this type of cancer. This finding was in accordance with a previous study demonstrating that IL-18 was downregulated in CRC (Pagès et al., 1999). However, no association between the IL-18 -607 C/A polymorphism and susceptibility to BC was observed in the present study population. This finding was not consistent with a previous study demonstrating that the IL-18 serum levels were notably increased in metastatic compared with non-metastatic BC patients. In addition, the authors suggested that IL-18 could contribute to doxorubicin resistance (Günel et al., 2002). Therefore, due to its dual roles in both drug resistance and tumor metastasis, IL-18 may serve as a target for BC therapy. It has been shown that IL-18, in synergy with IL-12, plays a key role in Th1 responses by upregulating IFN-γ, and exhibits anti-tumor activities via mediating the increase of NK cell activity and induction of tumor cell apoptosis (Srabović et al., 2011). Furthermore, the increased IL-18 levels serve a major role in PCa cell growth, invasion, and metastasis (Nong et al., 2013). Therefore, it has been suggested that the elevated secretion of IL-18BP by PCa cells indicates the effort of cancer cells to overcome immune surveillance (Fujita et al., 2011). In addition, IL-18 potentiates antitumor immunity in the tumor microenvironment via the innate and adaptive immune responses (Tse et al., 2011).

Although, CA 15-3, CA 19-9, CEA, and TPSA are still considered as excellent biomarkers for the early detection of BC, CRC and PCa, the levels of liver enzymes may serve as alternative indicators for the early detection of CRC and PCa. The findings of the present study did not support a significant association between the IL-18 -670 C/A polymorphism and the risk of BC, CRC and PCa. However, this polymorphism may be regarded as a risk factor for BC, CRC and PCa. Therefore, further studies with larger number of study participants from different ethnic groups should be performed to further investigate the diagnostic potential of the aforementioned biomarkers. The results of these studies may exhibit a beneficial clinical effect.

## References

[B1] Adamcova M, Popelova-Lencova O, Jirkovsky E (2016). Cardiac troponins--Translational biomarkers in cardiology: Theory and practice of cardiac troponin high-sensitivity assays. Biofactors.

[B2] Akinyemiju T, Sakhuja S, Waterbor J (2018). Racial/ethnic disparities in de novo metastases sites and survival outcomes for patients with primary breast, colorectal, and prostate cancer. Cancer Med.

[B3] Arends J, Bachmann P, Baracos V (2017). ESPEN guidelines on nutrition in cancer patients. Clin Nutr.

[B4] Blaes AH, Rehman A, Vock DM (2015). Utility of high-sensitivity cardiac troponin T in patients receiving anthracycline chemotherapy. Vasc Health Risk Manag.

[B5] Brown JC, Winters-Stone K, Lee A (2012). Cancer, physical activity, and exercise. Comprehensive Physiol.

[B6] Cao R, Farnebo J, Kurimoto M (1999). Interleukin-18 acts as an angiogenesis and tumor suppressor. Faseb J.

[B7] Cao R, Wang LP (2012). Serological diagnosis of liver metastasis in patients with breast cancer. Cancer Biol Med.

[B8] Diamandis EP (2014). Present and future of cancer biomarkers. Clin Chem Lab Med.

[B9] Field KM, Dow C, Michael M (2008). Part I: Liver function in oncology: biochemistry and beyond. Lancet Oncol.

[B10] Florido R, Lee AK, McEvoy JW (2019). Cancer Survivorship and Subclinical Myocardial Damage. Am J Epidemiol.

[B11] Frères P, Bouznad N, Servais L (2018). Variations of circulating cardiac biomarkers during and after anthracycline-containing chemotherapy in breast cancer patients. BMC Cancer.

[B12] Fu Y, Li H (2016). Assessing clinical significance of serum CA15-3 and carcinoembryonic antigen (CEA) levels in breast cancer patients: A Meta-Analysis. Med Sci Monit.

[B13] Fujita K, Ewing CM, Isaacs WB (2011). Immunomodulatory IL-18 binding protein is produced by prostate cancer cells and its levels in urine and serum correlate with tumor status. Int J Cancer.

[B14] Gaudreau PO, Stagg J, Soulières D (2016). The present and future of biomarkers in prostate cancer: Proteomics, genomics, and immunology advancements. Biomark Cancer.

[B15] Günel N, Coşkun U, Sancak B (2002). Clinical importance of serum interleukin-18 and nitric oxide activities in breast carcinoma patients. Cancer.

[B16] Hashemi SM, Arbabi N, Hashemi M (2017). Association between VDR Gene Polymorphisms (rs 1544410, rs 7975232, rs 2228570, rs 731236 and rs 11568820) and Susceptibility to Breast Cancer in a Sample of Southeastern Iranian Population. Int J Cancer Manage.

[B17] Hosseini-Baraftabi N, Zia-Jahromi N, Talebi A (2019). Comparison of interleukin 18 gene expression and its serum level between Iranian colorectal cancer (CRC) patients and healthy people. Biologia.

[B18] Jia Y, Zang A, Jiao S (2016). The interleukin-18 gene promoter -607 A/C polymorphism contributes to non-small-cell lung cancer risk in a Chinese population. Onco Targets Ther.

[B19] Kazarian A, Blyuss O, Metodieva G (2017). Testing breast cancer serum biomarkers for early detection and prognosis in pre-diagnosis samples. Br J Cancer.

[B20] Keshaviah A, Dellapasqua S, Rotmensz N (2007). CA15-3 and alkaline phosphatase as predictors for breast cancer recurrence: A combined analysis of seven. Int Breast Cancer Study Group Trials.

[B21] Li B, Wang F, Ma C (2019). Predictive value of IL-18 and IL-10 in the prognosis of patients with colorectal cancer. Oncol Lett.

[B22] Marcuello M, Vymetalkova V, Neves RPL (2019). Circulating biomarkers for early detection and clinical management of colorectal cancer. Mol Aspects Med.

[B23] Metwalli AR, Rosner IL, Cullen J (2014). Elevated alkaline phosphatase velocity strongly predicts overall survival and the risk of bone metastases in castrate resistant prostate cancer. Urol Oncol.

[B24] Ng L, Wan TM, Man JH (2017). Identification of serum miR-139-3p as a non-invasive biomarker for colorectal cancer. Oncotarget.

[B25] Ning S, Wei W, Li J (2018). Clinical significance and diagnostic capacity of serum TK1, CEA, CA 19-9 and CA 72-4 levels in gastric and colorectal cancer patients. J Cancer.

[B26] Nong S, Zhang Y, Cheng B (2013). Effect of interleukin-18 polymorphisms-607 and -137 on clinical characteristics of prostate cancer patients. Chin German J Clin Oncol.

[B27] Okamura R, Hasegawa S, Hida K (2017). The role of periodic serum CA19-9 test in surveillance after colorectal cancer surgery. Int J Clin Oncol.

[B28] Pagès F, Berger A, Henglein B (1999). Modulation of interleukin-18 expression in human colon carcinoma: consequences for tumor immune surveillance. Int J Cancer.

[B29] Park S, Cheon S, Cho D (2007). The dual effects of interleukin-18 in tumor progression. Cell Mol Immunol.

[B30] Qader G, Aali M, Amen KM (2020). The status of cancer publications in the Kurdistan region of Iraq. J Cancer Policy.

[B31] Rao SR, Snaith AE, Marino D (2017). Tumour-derived alkaline phosphatase regulates tumour growth, epithelial plasticity and disease-free survival in metastatic prostate cancer. Br J Cancer.

[B32] Romero Otero J, Garcia Gomez B, Campos Juanatey F (2014). Prostate cancer biomarkers: an update. Urol Oncol.

[B33] Saif MW, Alexander D, Wicox CM (2005). Serum alkaline phosphatase level as a prognostic tool in colorectal cancer: A Study of 105 patients. J Appl Res.

[B34] Shin JK, Kim HC, Lee WY (2019). High preoperative serum CA 19-9 levels can predict poor oncologic outcomes in colorectal cancer patients on propensity score analysis. Ann Surg Treat Res.

[B35] Singh AK, Pandey A, Tewari M (2013). Advanced stage of breast cancer hoist alkaline phosphatase activity: risk factor for females in India. Biotech.

[B36] Srabović N, Mujagić Z, Mujanović-Mustedanagić J (2011). Interleukin 18 expression in the primary breast cancer tumour tissue. Med Glas (Zenica).

[B37] Taheri M, Hashemi-Shahri SM, Hamzehnejadi M (2012). Lack of association between interleukin-18 -607 C/A gene polymorphism and pulmonary tuberculosis in Zahedan, Southeast Iran. Prague Med Rep.

[B38] Tse BW, Russell PJ, Lochner M (2011). IL-18 inhibits growth of murine orthotopic prostate carcinomas via both adaptive and innate immune mechanisms. PLoS One.

[B39] Youlden DR, Cramb SM, Yip CH (2014). Incidence and mortality of female breast cancer in the Asia-Pacific region. Cancer Biol Med.

[B40] Zhao E, Cui D, Yuan L (2012). MDM2 SNP309 polymorphism and breast cancer risk: a meta-analysis. Mol Biol Rep.

